# Effects of hyperbaric air exposure with oxygen breathing during decompression on the exhaled volatile organic compounds

**DOI:** 10.3389/fphys.2026.1893301

**Published:** 2026-09-08

**Authors:** Haoze Zheng, Chenglong Li, Wenwu Liu, Kun Wang, Jia He, Shifeng Wang

**Affiliations:** 1 Department of Diving and Hyperbaric Medicine, Naval Medical Center, Naval Medical University, Shanghai, China; 2 School of Medicine, Shanghai University, Shanghai, China

**Keywords:** diving, exhaled breath, hyperbaric exposure, oxygen-breathing decompression, volatile organic compounds

## Abstract

**Background:**

This study aimed to investigate changes in exhaled volatile organic compounds (VOCs) in divers exposed to hyperbaric air.

**Methods:**

A total of 17 adult divers underwent two sessions of hyperbaric air exposure at 30 m (400 kPa), separated by a one-week interval. In the first exposure, the divers were decompressed with hyperbaric air alone; in the second exposure, they breathed 100% oxygen for 30 min when decompressed to 18 m. The composition and concentration of VOCs in exhaled breath were measured before exposure, at the initiation of decompression, and after exposure.

**Results:**

The results showed that, during decompression with air, several olefins and alkanes, such as 1-heptene and n-heptane, increased after hyperbaric exposure. In contrast, during decompression with oxygen, alkanes, such as 2,2-dimethylbutane, and oxygenated compounds, such as acetone and isopropanol, decreased. Dimethyl sulfide decreased under both conditions. Compared with VOCs immediately before decompression, acrolein and isoprene increased significantly after exposure under both conditions; several alkanes, ketones, and olefins, including n-heptane, 2-butanone, and 1-heptene, decreased significantly during decompression with air; dimethyl sulfide increased significantly, and carbon disulfide showed an upward trend in the decompression with oxygen group after hyperbaric exposure. The fold change in peroxidation-related olefins and alkanes was markedly higher, and the fold change in carbon disulfide tended to increase in the decompression with oxygen group compared with the decompression with air group.

**Conclusion:**

Oxygen breathing during decompression appears to delay or alter the clearance of certain exhaled alkanes, ketones, and olefins while promoting the generation of sulfur-containing compounds.

## 1 Introduction

During hyperbaric exposure, divers routinely breathe gases with the partial pressure of oxygen (PO_2_) significantly higher than that under normobaric conditions. In addition, during decompression in diving or hyperbaric treatment, divers often breath gas mixtures with high PO_2_ or even pure oxygen at shallower depths, which may not only shorten the decompression time but also enhance safety and prevent the occurrence of decompression sickness (DCS). However, oxygen possesses oxidative capability and may cause an imbalance between oxidation and antioxidation in the body when one is exposed to an elevated PO_2_, leading to oxidative stress and subsequent adverse physiological effects (Wingelaar et al., 2017; Amarelle et al., 2021). The lung acts as the first-line defense at the environmental interface and is therefore susceptible to the detrimental effects of hyperoxia. Consequently, identifying non-invasive methods to monitor hyperoxic lung injury during and after hyperbaric exposure remains a critical focus of diving medicine research.

Damage to lung tissues is often accompanied by alterations in the composition of exhaled breath. The analysis of exhaled breath offers distinct advantages, including ease of implementation, non-invasiveness, and suitability for dynamic monitoring, and therefore an increasing number of studies have applied the analysis of exhaled breath in the clinical diagnosis and assessment of diseases, especially pulmonary disorders. Some recent studies have demonstrated that changes in the concentrations of nitric oxide (NO) and related metabolites in exhaled breath and exhaled breath condensate (EBC) are associated with hyperoxic lung injury (HLI) induced by high PO_2_ exposure (Fothergill and Gertner, 2023), as well as with the amount of bubbles formed following decompression in a dive (Pontier et al., 2014). With advances in research, “exhaled breath metabolomics” has provided novel insights into the non-invasive monitoring of respiratory diseases (Bos et al., 2014). Evidence shows that exposure to hyperoxia leads to increased “leakage” of reactive oxygen species (ROS) from the mitochondrial electron transport chain. When the amount of ROS accumulated in cells exceeds the scavenging capacity of the endogenous antioxidative system, these ROS may attack polyunsaturated fatty acids (PUFAs) within cellular membranes, triggering lipid peroxidation (LPO) (Jeong et al., 2025; Zhou et al., 2025). The metabolic byproducts of LPO, such as short-chain alkanes (e.g., ethane, n-pentane) and specific aldehydes and ketones, are classified as volatile organic compounds (VOCs). These molecules possess high diffusivity, enabling them to rapidly cross the blood-gas barrier and be excreted via exhaled breath. Gas chromatography–mass spectrometry (GC-MS), with its high sensitivity (detection limits at ppb or even ppt level) and high resolution, allows for the precise and dynamic detection of VOCs in the exhaled breath of divers, and thus these VOCs may serve as crucial biomarkers reflecting oxidative damage (de Jong et al., 2023).

Studies have shown that hyperbaric hyperoxia may induce LPO and inflammatory responses in alveolar membranes, e.g., phosphatidylcholine. Notably, alterations in specific VOCs, such as methylated alkanes and aldehydes, are detectable prior to the presence of clinical symptoms or declines in traditional pulmonary function test (PFT) parameters (van Ooij et al., 2014; Wingelaar et al., 2019a). This phenomenon has been validated in divers after different types of diving, including diving with pure oxygen, deep diving with heliox, and diving with closed-circuit rebreather (Wingelaar et al., 2019b; Wingelaar et al., 2020; de Jong et al., 2023). To systematically identify and standardize these metabolic signatures, researchers have integrated multiple GC-MS datasets to construct a “VAPOR profile” comprising 29 hyperbaric hyperoxia-specific VOCs (de Jong et al., 2022a). Although some specific VOCs have the potential to serve as characteristic biomarkers, few studies have analyzed VOCs in exhaled breath samples collected from individuals during hyperbaric exposure. Furthermore, whether oxygen breathing during decompression influences VOC profiles remains unreported.

In this study, we investigated the VOC profiles of exhaled breath in divers exposed to hyperbaric air at 30m. Exhaled breath samples were collected before exposure, immediately before decompression, and after chamber exit. The VOC profiles were compared between divers breathing air during decompression and those breathing pure oxygen at 18m for 30min during decompression. This study aimed to identify non-invasive biomarkers that can be used to assess the effects of hyperbaric air exposure on the lung, as well as the effects of decompression with oxygen on the lung.

## 2 Materials and methods

### 2.1 Study subjects

Seventeen male divers were recruited in October 2025. All participants met the National Medical Standards for Occupational Divers of the People’s Republic of China (GB 20827-2025). Volunteers had no recent respiratory infections and did not undergo any hyperbaric exposure within one week before the study. Strenuous physical activity and alcohol consumption were prohibited within 24h before the hyperbaric exposure. On the day of hyperbaric exposure, all participants underwent physical examination; none took any medications or supplements before exposure. All participants consumed a standardized diet on the day of exposure to avoid interference from irritant foods (e.g., pepper, garlic) and they were asked to abstain from smoking for at least 12h before study. Additionally, intake of any substances other than water was prohibited within one hour prior to sample collection to prevent contamination of the exhaled breath samples. This study was approved by the Ethics Committee of the Naval Medical University.

### 2.2 Hyperbaric exposure

Hyperbaric exposures were conducted in a dry chamber for hyperbaric exposure (1.0 MPa; Yantai Hongyuan Oxygen Industry Co., Ltd.) at the Naval Medical Center of the Chinese People’s Liberation Army. During the experiment, the compressed air breathed by the participants inside the chamber was subjected to two-stage purification and medical-grade pure oxygen was used for breathing. All the participants received two hyperbaric exposures separated by one week. In the first week, participants received hyperbaric air exposure with decompression by breathing air; in the second week, participants underwent hyperbaric air exposure, but they breathed pure oxygen at 18m for 30min during decompression. The specific protocols were as follows: in the air-breathing decompression group, participants were compressed with compressed air to 400 kPa (30m) at a rate of 10 m/min. The pressure was maintained for 20min, and the participants breathed air in the chamber. Then, they were depressurized to 12m (3min), 9m (12min), 6m (23min) and 3m (20min), and then surfaced; in the oxygen-breathing decompression group, participants were pressurized to 400 kPa, and then de-pressurized 20min later, at 18m, participants were asked to breathe oxygen via a mask for 30min, and then they were depressurized to 9m (8min), 6m (8min) and 3m (4min), and then surfaced.

### 2.3 Collection of exhaled breath samples

Exhaled breath samples were collected before exposure (Pre), immediately before decompression (Mid), and after chamber exit (Post). To minimize background interference, ambient air samples were collected outside the chamber to characterize the background concentrations corresponding to the Pre and Post breath samples, whereas a chamber air blank sample was collected at Mid to characterize the background VOC concentrations inside the chamber. The concentrations measured in the corresponding ambient or chamber air samples were subtracted from those measured in the exhaled breath samples at each time point. Exhaled breath samples were collected using 1-L Tedlar^®^ gas sampling bags (Dalian Delin Gas Packaging Co., Ltd., China). Before sampling, each bag was flushed with high-purity nitrogen (purity ≥ 99.99%) and then evacuated to remove residual nitrogen. Participants were instructed to inhale deeply to total lung capacity and exhale at a constant flow rate. The initial 150–300 mL of exhaled gas, representing the dead space mixture gas, was discarded, and the subsequent end-expiratory fraction, representing alveolar gas, was collected into the sampling bag. The bag was sealed immediately after collection. All samples were stored at 4°C in the dark, and samples were analyzed within 12h.

### 2.4 Detection of VOCs

#### 2.4.1 Sample collection and pre-treatment

Exhaled breath (500 mL) was injected via a VOC adsorption tube sampler (EM-500; AMAE Instruments Co., Ltd., China) at a flow rate of 250 mL/min. Then, the exhaled breath was pumped through stainless-steel adsorption tubes filled with Tenax-TA adsorbent material (Lichuang Application Company, China) for VOC enrichment. Prior to sampling, all thermal desorption tubes were pre-conditioned using a thermal desorption tube conditioner (Lichuang Application Company, China) to remove background contaminants. For conditioning, the tubes were heated to 200°C and purged continuously with helium at 1 mL/min (Smith et al., 2022).

#### 2.4.2 Thermal desorption-gas chromatography-mass spectrometry analysis

The adsorbed samples were analyzed using a portable GC-MS system (QitVenture 1, Ningbo Pangu Technology Co., Ltd.). Loaded adsorption tubes were placed into the thermal desorption inlet system, where compounds were desorbed and analyzed under optimized chromatographic and spectrometric conditions.

Chromatographic separation was achieved using a temperature gradient program: the oven temperature was first maintained at 60°C for 1min, ramped to 80°C at 20°C/min, and then increased to 230°C at 60°C/min, was maintained for 0.5min. The injection port temperature was set to 130°C, and the ten-port valve temperature was maintained at 50°C. Helium (99.999% purity) was used as the carrier gas at a constant flow rate (0.2 mL/min) (Liu et al., 2024) with a split ratio of 100:1.

Mass spectrometric detection was configured with an electron impact ionization (EI) source. The ion trap temperature was set at 50°C, and the transfer line temperature was maintained at 150°C. Data were acquired in full scan mode over a mass-to-charge (m/z) range of 40–300 amu, with an ionization energy of 70 eV and a scan duration of 5min. Instrument control and data acquisition were managed using the built-in GC-MS software (Zhang et al., 2024).

#### 2.4.3 Data processing and identification

Raw data from GC-MS were processed using the built-in software, including baseline correction, noise reduction, and peak identification. Features with a peak area greater than 100 were retained for further analysis. The mass spectra and retention indices were compared with those from the National Institute of Standards and Technology (NIST) mass spectral library to identify compounds.

#### 2.4.4 Statistical analysis

Data with normal distribution are presented as mean ± standard deviation, whereas concentrations of VOCs with non-normal distributions are expressed as median (interquartile range). All statistical analyses were performed using Python (version 3.13.5). Data preprocessing and visualization were conducted utilizing the Pandas, NumPy, SciPy, Statsmodels, Matplotlib, and Seaborn libraries. Comparisons of results among the three time points were performed using the Friedman test. For *post-hoc* pairwise comparisons between time points, the Wilcoxon signed-rank test was applied. To quantify the magnitude of observed changes independently of sample size, the rank-biserial correlation coefficient (r_rb_) was calculated as the primary effect size. The false discovery rate (FDR) was controlled using the Benjamini-Hochberg procedure. In all statistical analyses, two-sided P < 0.05 was considered statistically significant. For all pairwise comparisons, 95% confidence intervals (CIs) for r_rb_ were estimated using the bias-corrected and accelerated (BCa) bootstrap method with 5,000 resamples to mitigate bias associated with small sample sizes.

## 3 Results

### 3.1 General information

A total of 17 male divers completed the study. The demographic and anthropometric characteristics of these participants are summarized as follows: mean age was 25.0 ± 2.2 years, mean height was 172.7 ± 5.3cm, mean weight was 70.5 ± 7.0kg, and mean body mass index (BMI) was 23.6 ± 2.4 kg/m².

Following data preprocessing and spectral matching against the NIST library, 27 common ambient air pollutants and endogenous volatile metabolites were initially screened. Based on previous reports (de Jong et al., 2022), ubiquitous atmospheric contaminants and potential chamber-derived contaminants were excluded. The excluded compounds included chlorofluorocarbons (e.g., trichlorofluoromethane), solvents (e.g., tetrahydrofuran, dichloromethane, 1,2-dichloroethane), aromatic hydrocarbons (e.g., ethylbenzene), and halogenated benzenes (e.g., 1,2-dichlorobenzene). Consequently, 14 compounds exhibiting significant changes in the content were identified and processed for further analysis (**Table 1**).

**Table 1:** Major exhaled VOCs with significant alteration in the whole exposure phase.

Compounds	Retention time (Min)	Cas no.	Quantitative lon (M/Z)
1,2-Pentadiene	0.3500	591-95-7	67
1-Heptene	0.9833	592-76-7	56,41
1-Pentene/(E/Z)-2-Pentene	0.2683	109-67-1/646-04-8/627-20-3	55
2-Butanone	0.4583	78-93-3	72
Carbon Disulfide	0.3300	75-15-0	76
Isoprene	0.2783	78-79-5	67,68
n-Heptane	0.9667	142-82-5	57,71
Methylcyclohexane	1.3117	108-87-2	83
Dimethyl Sulfide	0.2817	75-18-3	62,47
2,2-Dimethylbutane	0.4517	75-83-2	71,70
Acetone	0.2550	67-64-1	58,43
Methyl Acetate	0.2700	79-20-9	74
(E/Z)-2-Butene	0.2517	624-64-6/590-18-1	41,56
Acrolein	0.2517	107-02-8	56,55

### 3.2 Changes in exhaled VOCs after hyperbaric exposure with air alone

**Figure 1:**
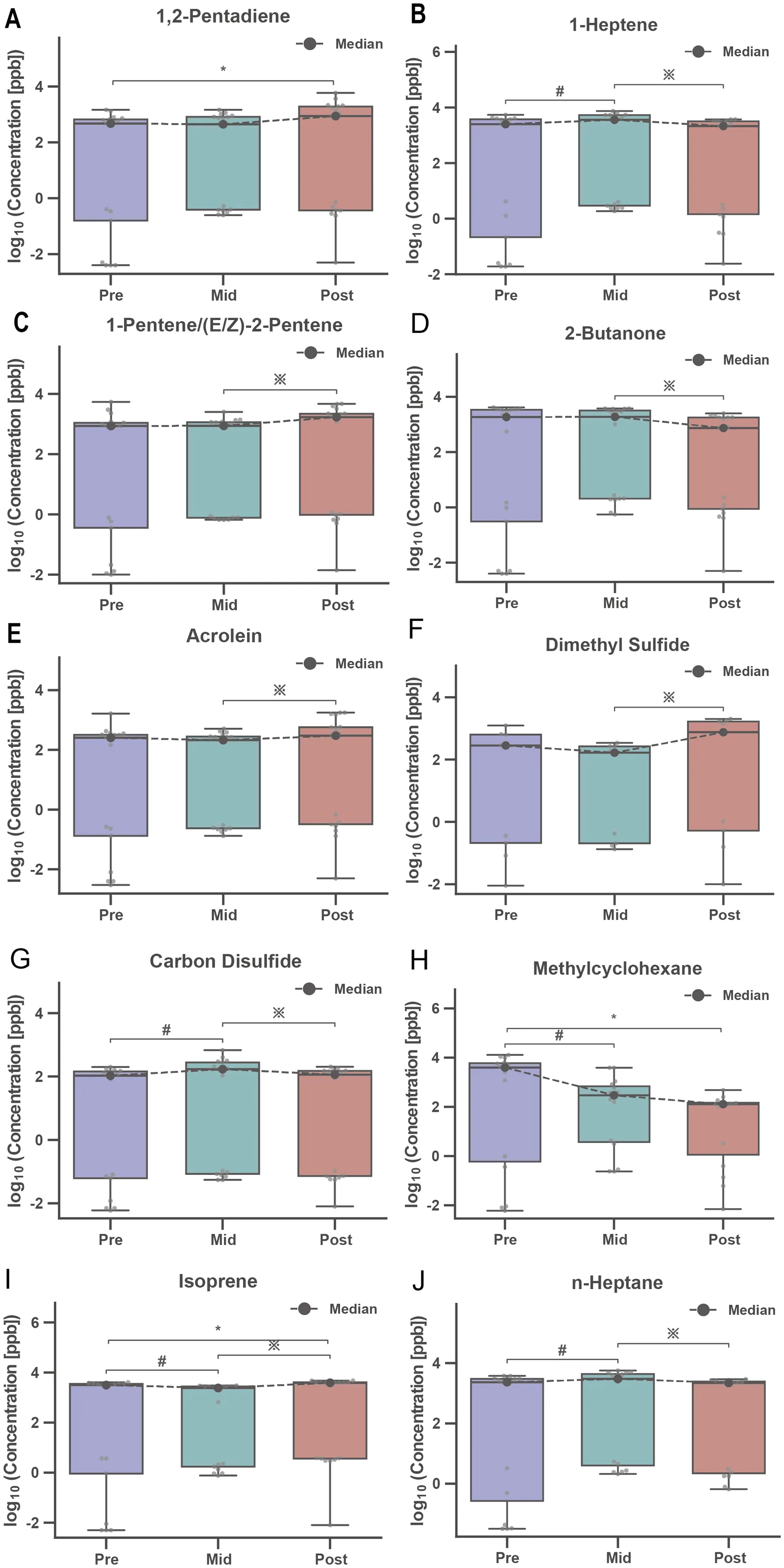
Major VOCs of exhaled breath with significant change or trends in content at different time points in diver with hyperbaric air exposure. **(A)** 1,2-Pentadiene; **(B)** 1-Heptene; **(C)** 1-Pentene/(E/Z)-2-Pentene; **(D)** 2-Butanone; **(E)** Acrolein; **(F)** Dimethyl Sulfide; **(G)** Carbon Disulfide; **(H)** Methylcyclohexane; **(I)** Isoprene; and **(J)** n-Heptane. *P< 0.05 *vs* pre-exposure (Pre); ^#^ *P*< 0.05 *vs* mid-exposure (Mid); ^※^ *P*< 0.05 *vs* post-exposure (Post). Box plots depict medians (center lines), interquartile ranges (IQR, boxes), and 1.5 × IQR whiskers; outliers are shown as individual dots.

Repeated-measures analysis using the Friedman test showed that the concentrations of eight exhaled VOCs changed significantly across three time points in the hyperbaric air exposure group (**Figure 1**). These compounds included 1,2-pentadiene (P=0.023), 1-heptene (P=0.0005), 1-pentene/(E/Z)-2-pentene (P=0.009), 2-butanone (P=0.007), carbon disulfide (P=0.007), isoprene (P=0.0001), n-heptane (P=0.0003), and methylcyclohexane (P=0.014).

The overall Friedman test for dimethyl sulfide across three time points did not reach statistical significance (P=0.066). Therefore, FDR-corrected *post-hoc* pairwise comparisons were further performed using the Wilcoxon signed-rank test. Dimethyl sulfide showed a decreasing trend from Pre to Mid (FDR-adjusted P=0.064; median concentration:284.984 *vs*. 0.489; r_rb_ = −0.782, 95% CI [−1, −0.273]). In contrast, its concentration increased significantly from Mid to Post (FDR-adjusted P=0.033; median concentration: 0.489 *vs*. 754.679; r_rb_ = 0.821, 95% CI [0.385, 1]). Although an increasing trend was also observed from Pre to Post, no significant difference was observed after FDR correction (FDR-adjusted P=0.128; median concentration: 284.984 *vs*. 754.679; r_rb_ = 0.582, 95% CI [0.011, 1]).

Compared with pre-exposure (Pre), the concentrations of 1-heptene (P=0.028), carbon disulfide (P=0.013), and n-heptane (P=0.008) were significantly increased immediately before decompression (Mid) (**Figures 1B, G, J**). In contrast, the concentrations of isoprene (P=0.033) and methylcyclohexane (P=0.028) were significantly decreased at Mid compared with Pre (**Figures 1I, H**). In addition, trans-/cis-2-butene showed an increasing trend from Pre to Mid, and this change did not reach statistical significance after FDR correction (FDR-adjusted P=0.052; median concentration: 1097.8 *vs*. 1934.87; r_rb_ = 0.634, 95% CI [0.150, 1]) (**Figure 1C**).

Compared with pre-exposure (Pre), the concentrations of 1,2-pentadiene (P=0.012) and isoprene (P=0.003) were significantly increased after hyperbaric exposure (Post) (**Figures 1A, H**), whereas the concentration of methylcyclohexane was significantly decreased (P=0.012) (**Figure 1G**). In addition, 1-pentene/(E/Z)-2-pentene showed an increasing trend from Pre to Post, although this change did not reach statistical significance after FDR correction (FDR-adjusted P=0.052; median concentration: 859.415 *vs*. 1686.347; r_rb_ = 0.634, 95% CI [0.163, 1]) (**Figure 1C**).

Compared with before decompression (Mid), the concentrations of 1-pentene/(E/Z)-2-pentene (P=0.033) and isoprene (P=0.002) were significantly increased after hyperbaric exposure (Post) (**Figures 1C, H**). In contrast, the concentrations of 1-heptene (P=0.002), 2-butanone (P=0.012), carbon disulfide (P=0.012), and n-heptane (P=0.017) were significantly decreased at Post compared with Mid (**Figures 1B, D, F, I**). In addition, 1,2-pentadiene showed an increasing trend from Mid to Post, even after FDR correction (FDR-adjusted P=0.093; median concentration: 444.85 *vs*. 871.915; r_rb_ = 0.556, 95% CI [0.033, 0.895]) (**Figure 1A**). Methylcyclohexane showed a decreasing trend after FDR correction (FDR-adjusted P=0.050; median concentration: 198.788 *vs*. 106.874; r_rb_ = −0.647, 95% CI [−0.935, −0.203]) (**Figure 1G**).

### 3.3 Changes in exhaled VOCs during oxygen-breathing decompression

**Figure 2:**
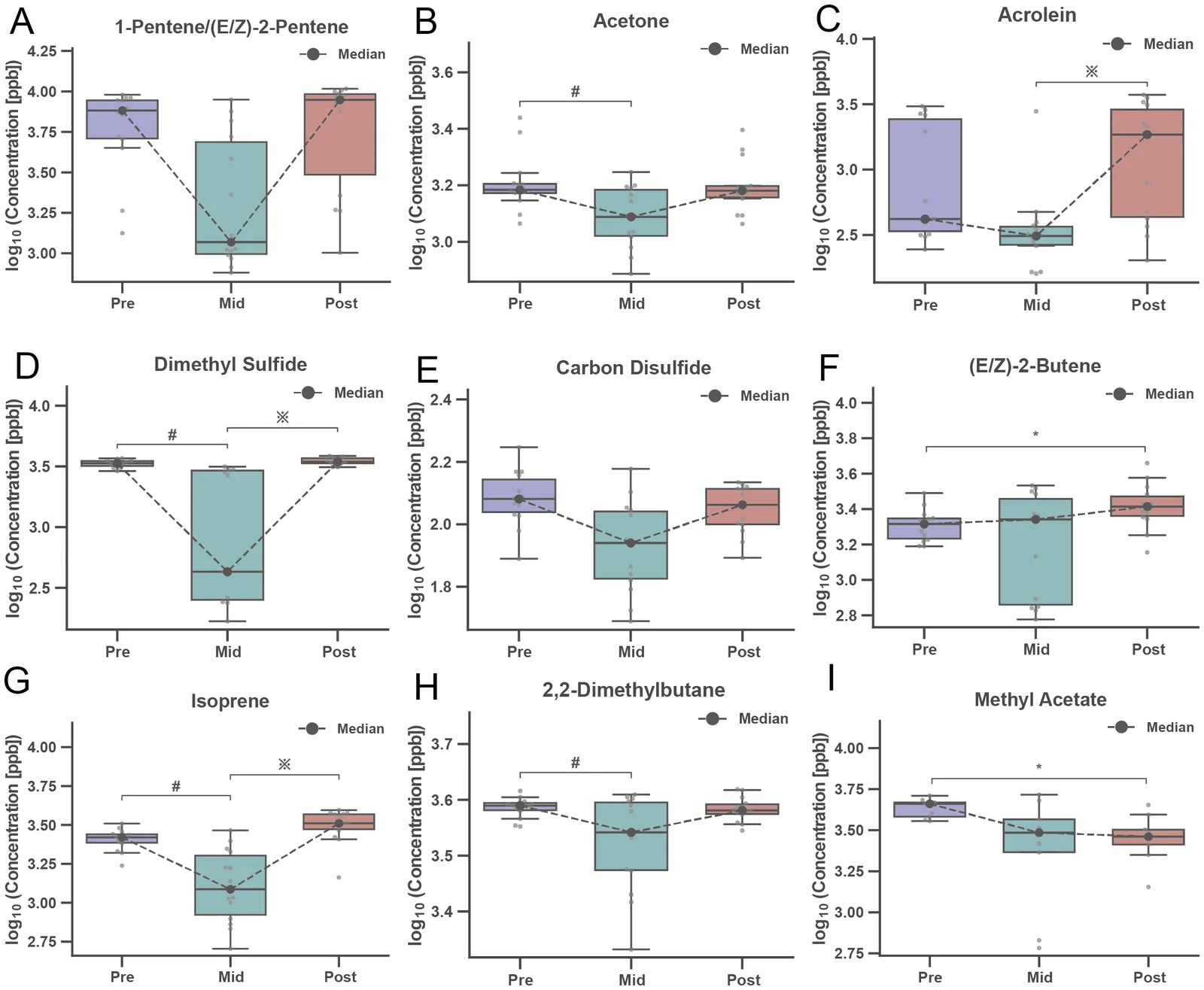
Major VOCs of exhaled breath with significant change or trends in content at different time points in divers experiencing decompression with oxygen-breathing. **(A)** 1-Pentene/(E/Z)-2-Pentene; **(B)** Acetone; **(C)** Acrolein; **(D)** Dimethyl Sulfide; **(E)** Carbon Disulfide; **(F)** (E/Z)-2-Butene; **(G)** Isoprene; **(H)** 2,2-Dimethylbutane; and **(I)** Methyl Acetate. *P <0.05 *vs* before exposure (Pre); ^#^*P* <0.05 *vs* immediately before decompression (Mid); ^※^*P* <0.05 *vs* after exposure (Post). VOC concentrations were log_10_-transformed for visualization, whereas statistical analyses were performed using the original concentration values. Box plots depict medians (center lines), interquartile ranges (IQR, boxes), and 1.5 × IQR whiskers; outliers are shown as individual dots.

Repeated-measures analysis using the Friedman test showed that the concentrations of seven exhaled VOCs changed significantly across the three time points in the oxygen-breathing decompression group (**Figure 2**). These compounds included 1-pentene/(E/Z)-2-pentene (P=0.002), acrolein (P=0.0103), acetone (P=0.017), dimethyl sulfide (P=0.00008), carbon disulfide (P=0.030), trans-/cis-2-butene (P=0.011), and isoprene (P=0.00007).

The overall Friedman tests did not show significant differences in 2,2-dimethylbutane and methyl acetate across three time points, which might be ascribed to the relatively small sample size (n = 17). Therefore, FDR-corrected *post-hoc* pairwise comparisons were further performed using the Wilcoxon signed-rank test as exploratory analyses. For 2,2-dimethylbutane, a potential decrease was observed from Pre to Mid (FDR-adjusted P=0.024; median concentration: 3882.530 *vs*. 3480.990; r_rb_ = −0.867, 95% CI [−1.000, −0.524]), followed by a tendency toward recovery from Mid to Post (FDR-adjusted P=0.142; median concentration: 3480.990 *vs*. 3802.790; r_rb_ = 0.600, 95% CI [−0.048, 0.887]). For methyl acetate, a potential decrease was observed from Pre to Post (FDR-adjusted P=0.048; median concentration: 4210.010 *vs*. 3055.320; r_rb_ = −0.767, 95% CI [−0.983, −0.333]).

Compared with Pre, the concentrations of acetone (P=0.024), dimethyl sulfide (P=0.012), and isoprene (P=0.024) were significantly decreased at Mid (**Figures 2B, D, G**). In addition, 1-pentene/(E/Z)-2-pentene showed a decreasing trend from Pre to Mid (P=0.097; median concentration: 7622.479 *vs*. 1176.586; r_rb_ = −0.695, 95% CI [−1.000, −0.219]) (**Figure 2A**), as did acrolein (P=0.137; median concentration: 418.526 *vs*. 311.132; r_rb_ = −0.619, 95% CI [−1.000, −0.105]) (**Figure 2C**), carbon disulfide (FDR-adjusted P=0.081; median concentration: 120.517 *vs*. 87.136; r_rb_ = −0.733, 95% CI [−1.000, −0.295]) (**Figure 2E**) and isopropanol (FDR-adjusted P=0.127; median concentration: 1218.540 *vs*. 987.650; r_rb_ = −0.638, 95% CI [−0.943, −0.086]) (**Supplementary Figure 2**).

Compared with Pre, the concentration of trans-/cis-2-butene was significantly increased at Post (P=0.024) (**Figure 2F**). Dimethyl sulfide showed an increasing trend from Pre to Post (P=0.106; median concentration: 3329.313 *vs*. 3439.639; r_rb_ = 0.692, 95% CI [0.187, 1.000]) (**Figure 2D**), as did isoprene (P=0.106; median concentration: 2634.139 *vs*. 3237.589; r_rb_ = 0.650, 95% CI [0.167, 1.000]) (**Figure 2G**).

Compared with Mid, the concentrations of acrolein (P=0.027), dimethyl sulfide (P=0.012), and isoprene (P=0.009) were significantly increased at Post (**Figures 2C, D, G**). Moreover, 1-pentene/(E/Z)-2-pentene showed an increasing trend from Mid to Post (FDR-adjusted P=0.070; median concentration: 1176.586 *vs*. 8635.467; r_rb_ = 0.752, 95% CI [0.333, 1.000]) (**Figure 2A**), and carbon disulfide also showed an increasing trend from Mid to Post (FDR-adjusted P=0.142; median concentration: 87.136 *vs*. 115.271; r_rb_ = 0.600, 95% CI [0.067, 0.943]) (**Figure 2E**).

### 3.4 Comparison of VOC changes between the oxygen-breathing and air-breathing decompression

To evaluate the effect of oxygen breathing during decompression, the Mid-to-Post relative change in exhaled VOC concentrations was calculated for each participant as (Post − Mid)/Mid and compared between two groups using the paired Wilcoxon signed-rank test. From Mid to Post, the median relative changes under the air-breathing and oxygen-breathing conditions were −47.38% and 15.06% respectively for 1-heptene, −29.48% and 51.14% respectively for carbon disulfide, 55.81% and 330.84% respectively for 1-pentene/(E/Z)-2-pentene, −46.04% and −4.96% respectively for n-heptane, and 57.65% and 462.60% respectively for acrolein. These five compounds showed significant different between groups based on the unadjusted P values, with relatively large positive <equation_for_insert/> point estimates (0.626–0.758); however, no significant difference was observed after FDR correction. The relative change in 2-butanone was −52.56% under the air-breathing condition and −16.11% under the oxygen-breathing condition, with a relatively large effect-size estimate (<equation_for_insert/>, 95% CI [−0.187, 0.912]), although there was no significant difference (unadjusted P=0.1099; FDR-adjusted P=0.3296) (**Table 2**).

**Table 2:** Selected VOCs showing nominal between-condition differences or large effect-size estimates in Mid-to-Post relative changes.

Compounds	*P* value	BH-FDR-adjusted *P* value	Rank-biserial correlation r_rb_	95% CI for r_rb_
1-Heptene	0.0134	0.2051	0.758	0.081–0.978
Carbon disulfide	0.0171	0.2051	0.736	0.143–0.978
1-Pentene/(E/Z)-2-pentene	0.0398	0.2297	0.648	0.033–0.934
n-Heptane	0.0425	0.2297	0.667	−0.026–1.000
Acrolein	0.0479	0.2297	0.626	0.011–0.934
2-Butanone	0.1099	0.3296	0.516	−0.187–0.912

Mid-to-Post relative changes were compared between the two decompression conditions using the paired Wilcoxon signed-rank test.

## 4 Discussion

This study investigated dynamic changes in exhaled VOCs in 17 divers during pressurization to 400 kPa and subsequent decompression and compared the Mid-to-Post changes in VOCs between the air-breathing decompression and oxygen-breathing decompression conditions. After collection, the exhaled breath samples were stored at 4°C and analyzed within 12h. Previous studies have shown that this storage and analytical protocol can maintain recovery rates above 90% for most target gases (He et al., 2024).

Studies have demonstrated that changes in specific exhaled VOCs, primarily methylated alkanes and aldehydes, can be detected after hyperbaric hyperoxic exposure before the onset of clinical symptoms or measurable deterioration in pulmonary function test parameters (van Ooij et al., 2014; Wingelaar et al., 2019a). This phenomenon has been observed under several exposure conditions, including oxygen diving, deep heliox diving, closed-circuit rebreather diving, and hyperbaric treatment for decompression sickness (Wingelaar et al., 2019b; Wingelaar et al., 2020; de Jong et al., 2023). Exhaled VOC profiles may vary according to the specific hyperbaric exposure protocol. Compared with diving exposures, the VOC changes observed after routine hyperbaric oxygen therapy or treatment using U.S. Navy Treatment Table 6 appear to be relatively mild, with some methylated alkanes even showing decreasing trends (Wingelaar et al., 2019b; de Jong et al., 2022b).

The present study showed that changes in several alkanes, alkenes, oxygenated compounds, and sulfur-containing compounds were observed immediately before decompression (Mid). During decompression, the changes in VOCs of exhaled breath were different between groups. The paired analysis between groups showed significant differences in the Mid-to-Post relative changes of 1-heptene, carbon disulfide, 1-pentene/(E/Z)-2-pentene, n-heptane, and acrolein. 2-Butanone also showed a relatively large effect-size estimate but did not reach statistical significance. These compounds showed relatively large positive r_rb_ point estimates, suggesting that oxygen breathing during decompression may affect the generation, release, or clearance of certain VOCs.

Under the air-breathing decompression condition, 1-heptene and n-heptane increased by approximately 43.94% and 30.47%, respectively, from Pre to Mid after pressurization to 400 kPa, and both differences were statistically significant (P=0.028 and P=0.008, respectively). Carbon disulfide increased by approximately 60.26% (P=0.013). Isoprene and methylcyclohexane decreased significantly (both P < 0.05). These changes occurred before decompression began, indicating that hyperbaric air exposure altered the exhaled VOC profile. Straight-chain alkanes and medium-chain alkenes can be generated through the oxidative fragmentation of polyunsaturated fatty acids in pulmonary surfactant and cell membranes and may serve as nonspecific volatile products of lipid peroxidation (Gueraud et al., 2010; Hakim et al., 2012; Oyerinde et al., 2023). These volatile hydrocarbons have high lipophilicity and low boiling points, allowing them to cross the blood–gas barrier and enter the alveolar space, thereby reflecting reactive oxygen species-mediated lipid oxidation (Rattray et al., 2014). The increases in 1-heptene and n-heptane may be associated with mild early lipid peroxidation. However, the directions of change were not consistent across all VOCs, suggesting that changes in ventilation, circulatory perfusion, tissue solubility, and metabolic activity may also have contributed.

Under the oxygen-breathing decompression condition, the median Acetone significantly decreased by approximately 19.72% from Pre to Mid, and the significant difference remained after FDR correction. Dimethyl sulfide and isoprene also decreased significantly. Isopropanol decreased by approximately 18.95% although no marked difference was observed. Acrolein also showed a decreasing trend from Pre to Mid, and there was no significant difference. Because these changes occurred before oxygen breathing, they cannot be attributed to oxygen breathing during decompression and should instead be interpreted as early metabolic responses after pressurization to 400 kPa and as intra-individual variability between two groups. Across the entire exposure period, trans-/cis-2-butene increased significantly from Pre to Post, reflecting the combined effects of hyperbaric exposure and decompression rather than an isolated Pre-to-Mid response.

In the complete paired dataset used for the between-condition analysis, distinct Mid-to-Post patterns were observed under the two decompression conditions. The median relative change in 1-heptene was −47.38% during air-breathing decompression and 15.06% during oxygen-breathing decompression, whereas carbon disulfide decreased by 29.48% under the air-breathing condition but increased by 51.14% under the oxygen-breathing condition. The median relative change in 1-pentene/(E/Z)-2-pentene was 55.81% during air-breathing decompression and 330.84% during oxygen-breathing decompression. Acrolein also increased under both conditions, with median relative changes of 57.65% and 462.60%, respectively. In contrast, n-heptane decreased under both conditions, but the reduction was less pronounced during oxygen-breathing decompression (−46.04% vs. −4.96%). Similarly, 2-butanone decreased by 52.56% during air-breathing decompression and by 16.11% during oxygen-breathing decompression. Paired Wilcoxon signed-rank tests showed significant differences between groups in the 1-heptene, carbon disulfide, 1-pentene/(E/Z)-2-pentene, n-heptane, and acrolein, with unadjusted P values ranging from 0.0134 to 0.0479 and relatively large positive rank-biserial correlation coefficients. However, no significant differences were observed after Benjamini–Hochberg FDR correction. Although the difference in 2-butanone did not reach statistical significance, its effect-size estimate was relatively large. These suggest that oxygen breathing during decompression may influence the generation, release, or clearance of selected hydrocarbons, aldehydes, ketones, and sulfur-containing compounds. Nevertheless, because the CIs were relatively wide and no significant difference was observed after FDR correction. Therefore, these VOCs should be interpreted cautiously.

Acrolein was one of the most pronounced contrasts between groups. Its median Mid-to-Post relative change was 57.65% under air-breathing condition and 462.60% under oxygen-breathing condition. Acrolein increased significantly from Mid to Post within the oxygen-breathing condition, whereas the paired comparison between groups reached only nominal significance. Therefore, the large effect-size point estimate supports acrolein as a potential biological candidate, but it does not establish a confirmed oxygen-specific effect. The Mid-to-Post relative changes in 1-heptene (a pentene isomer) and n-heptane also showed nominal significance between groups with relatively large effect-size estimates. However, no marked difference was observed after FDR correction.

Acrolein can be generated through the cleavage of lipid peroxidation products derived from polyunsaturated fatty acids and through oxidative polyamine catabolism involving spermine oxidase and polyamine acetyltransferase. Acrolein can deplete intracellular antioxidants and induce protein carbonylation and mitochondrial dysfunction, thereby aggravating oxidative stress-mediated cellular injury (Sebela and Raskova, 2023; Bae et al., 2025). Breathing pure oxygen at 280 kPa for 30min during decompression may increase reactive oxygen species generation and disrupt redox homeostasis, thereby promoting the formation or release of acrolein. The link with lipid peroxidation remains tentative and warrants further validation using concurrent biochemical markers.

The acetone findings differed from those for acrolein. Acetone decreased significantly from Pre to Mid within the oxygen-breathing session, indicating that this change occurred before oxygen breathing during decompression. In the paired analysis between groups, the median Mid-to-Post relative changes were 1.16% under air-breathing condition and 23.81% under oxygen-breathing condition; however, the difference was not statistically significant (unadjusted P=0.1677; FDR-adjusted P=0.3354; r_rb_=0.451, 95% CI [−0.231, 0.868]). Acetone is primarily derived from fatty acid β-oxidation and ketone-body metabolism. Acetoacetate can undergo spontaneous decarboxylation to form acetone, which subsequently enters the circulation and is excreted through the lungs. Exhaled acetone may therefore reflect, to a certain extent, energy-substrate utilization and lipid metabolic status (Anderson, 2015). Its concentration may also be affected by nutritional status, basal metabolism, sympathetic nervous system activity, and individual metabolic variability. Acetone may serve as a potential marker of energy-substrate metabolism, although the present study did not reveal a significant difference between groups. Therefore, Acetone should be interpreted as a potential within-session marker of metabolic variation rather than an indicator of oxygen-mediated effect.

Isoprene decreased at Mid and recovered or increased at Post under both decompression conditions. Isoprene is one of the most abundant endogenous VOCs in human breath, and its origin may be associated with IDI2/DMAPP-related metabolism and skeletal muscle lipid–cholesterol metabolism (Sebela and Raskova, 2023). Isoprene is generally not regarded as a direct product of lipid peroxidation, and its exhaled concentration is influenced by cardiac output, peripheral perfusion, ventilation/perfusion matching, skeletal muscle activity, and tissue solubility. de Jong et al. observed an increase in exhaled isoprene 30min after U.S. Navy Treatment Table 6, followed by a decrease at 2h, indicating a marked time-dependent response (de Jong et al., 2022b). During pressurization, the participants remained seated and relatively inactive, which reduces the skeletal muscle activity; in addition, ambient pressure and breathing-gas density increased, which increases the tissue solubility. These factors eventually contribute to the decrease in isoprene at Mid. After decompression, restoration of peripheral circulation, reduction in ambient pressure, and decrease of tissue solubility may facilitate the release of isoprene stored in tissues during hyperbaric exposure. The similar pattern of isoprene changes under both conditions suggests that these changes primarily reflect circulatory and metabolic responses to hyperbaric exposure and decompression rather than an oxidative stress response specific to oxygen breathing during decompression.

Sulfur-containing compounds also exhibited different dynamic patterns under the two conditions. In the complete paired dataset, carbon disulfide decreased by 29.48% from Mid to Post under the air-breathing condition but increased by 51.14% under oxygen-breathing condition. The comparison showed significant difference and a relatively large effect-size estimate was observed although marked difference was not observed after FDR correction. This result should be interpreted cautiously and more studies are warranted for further confirmation. Dimethyl sulfide had a very low absolute concentration at Mid under air-breathing condition, which may magnify its subsequent relative rebound; its findings should therefore be interpreted using absolute concentrations, relative changes, and compound stability together. Hyperbaric hyperoxic exposure may alter the oxygen environment of anaerobic microorganisms on the mucosal surfaces of the gastrointestinal tract, oral cavity, and respiratory tract, thereby influencing the degradation and fermentation of sulfur-containing amino acids. Sulfur-containing VOCs may also be affected by endogenous sulfur amino acid metabolism, environmental background concentrations, and sample stability. Because neither the microbiota nor sulfur amino acid metabolism was assessed in the present study, it remains unclear whether the observed changes in carbon disulfide and dimethyl sulfide originated from microbial metabolism.

Some pairwise comparisons did not reach statistical significance after FDR correction, although the corresponding effect-size estimates indicated potentially meaningful changes. Under the air-breathing decompression condition, trans-/cis-2-butene increased from Pre to Mid (FDR-adjusted P=0.052, r_rb_ = 0.634, 95% CI [0.150, 1.000]). 1-pentene/(E/Z)-2-pentene increased from Pre to Post (FDR-adjusted P=0.052, r_rb_ = 0.634, 95% CI [0.163, 1.000]), and 1,2-pentadiene increased from Mid to Post (FDR-adjusted P=0.093, r_rb_ = 0.556, 95% CI [0.033, 0.895]). These findings indicate that some changes without significant difference after FDR correction nevertheless showed moderate-to-large effect-size estimates. The relatively small sample size may reduce statistical power and increase the risk of Type II errors. Because the CIs were relatively wide and multiple comparisons were performed, these findings require validation in larger cohorts.

Cronin et al. established a porcine model of pulmonary oxygen toxicity (POT) and found that a six-VOC breath score changed before deterioration in the PaO_2_/FiO_2_ ratio and the development of overt lung injury, with an approximate lead time of 36h (Cronin et al., 2019). These pathological and physiological findings support the potential use of VOCs as early-warning indicators of POT. Cronin et al. used prolonged normobaric​ hyperoxia exposure and identified predominantly methylated alkanes, whereas the present study involved short-term hyperbaric air exposure and oxygen breathing during decompression, with prominent changes involving straight-chain alkanes, alkenes, acrolein, acetone, and sulfur-containing compounds. The two studies differed in species, exposure duration, oxygen partial pressure, normobaric versus hyperbaric conditions, and statistical modeling approaches. The findings of Cronin et al. support the general potential of VOCs for early monitoring but do not demonstrate that acrolein or other VOCs identified in the present study can predict POT. The present findings are more appropriately interpreted as early, subclinical, and potentially reversible metabolic stress responses.

Fothergill et al. used LC–MS to analyze the exhaled breath condensate metabolome of 14 U.S. Navy-trained divers following 6.5h of hyperbaric oxygen exposure at 200 kPa and a nitrogen–oxygen exposure of the same pressure and duration and identified changes in lipid-derived and oxylipin-related metabolites (Fothergill et al., 2023). Their study examined non-volatile or low-volatility compounds in the aerosol–liquid phase of exhaled breath, whereas the present study used GC–MS to analyze gas-phase VOCs. Although the two studies differed in pressure and duration of hyperbaric exposure, sample type, and analytical platform, they provide complementary evidence from the non-volatile/low-volatility and volatile gas phases that hyperbaric hyperoxic exposure may alter oxidative lipid metabolism. Individual metabolites identified in the two studies should not be directly compared because of these methodological differences.

In addition to VOCs, circulating microparticles, neutrophil activation, inflammatory responses, and endothelial injury markers may be used to assess decompression stress (Thom et al., 2012; Zhang et al., 2017; Brett et al., 2019). VOCs may be suitable for reflecting rapid and dynamic gaseous metabolic responses, whereas circulating microparticles, inflammatory mediators, and endothelial injury markers may provide complementary information regarding vascular injury and immune activation. Integrating VOCs with EBC lipid metabolites, circulating microparticles, inflammatory markers, endothelial function, and pulmonary function testing may facilitate the development of a more comprehensive system for the monitoring of hyperbaric and decompression stress.

This study has several limitations. Only 17 participants were included. Although the design reduced the influence of inter-individual variability, the relatively small sample size limited statistical power and increased the risk of Type II errors, potentially preventing some metabolic changes with moderate or large effect sizes from reaching statistical significance after FDR correction. Post samples were collected only immediately after exposure, and therefore we failed to determinate the peak times, delayed-release patterns, and time to recovery of individual VOCs.

α,β-Unsaturated carbonyl compounds, such as acrolein, and some sulfur-containing compounds are susceptible to environmental sensitivity and sampling artifacts. Their stability in Tedlar^®^ bags is also lower than that of their corresponding saturated analogues. Storage at 4°C can reduce degradation but cannot completely prevent analyte loss. The absolute concentrations of these compounds should therefore be interpreted with caution. The present study primarily focused on relative changes within the same participants across different time points and between the two decompression conditions (Li et al., 2021).

## 5 Conclusion

This study demonstrated that pressurization to 400 kPa and subsequent decompression induced dynamic changes in exhaled VOC profiles. Changes in several alkanes, alkenes, oxygenated compounds, and sulfur-containing compounds were already evident before decompression, indicating early metabolic responses to hyperbaric air exposure. Paired comparisons of the Mid-to-Post relative changes identified nominal significant differences between groups in 1-heptene, carbon disulfide, 1-pentene/(E/Z)-2-pentene, n-heptane, and acrolein, with relatively large r_rb_ point estimates; however, no marked difference was noted after FDR correction. 2-Butanone also showed a relatively large effect-size estimate without nominal significance. These findings suggest that oxygen breathing during decompression may modify the post-decompression dynamics of selected VOCs, but the results require confirmation in larger, randomized studies. Exhaled VOC analysis may provide a non-invasive approach for characterizing early physiological responses to hyperbaric exposure and decompression. Together with the altered fold changes of several alkanes and alkenes, these findings support the involvement of oxidative stress, lipid peroxidation, and energy metabolic responses during hyperbaric exposure and oxygen-assisted decompression. Exhaled VOC analysis therefore provides a promising non-invasive approach for characterizing early physiological responses to hyperbaric and decompression stress in divers and may contribute to the development of objective indicators for individual oxygen sensitivity and early hyperoxic stress monitoring. Future studies should include larger cohorts, additional post-exposure sampling time points, and combined assessments of pulmonary function, EBC metabolomics, circulating inflammatory markers, and endothelial injury markers.

## Data Availability

The raw data supporting the conclusions of this article will be made available by the authors, without undue reservation.
