# Supplementary material for: Effects of hyperbaric air exposure with oxygen breathing during decompression on the exhaled volatile organic compounds
Source: Front Physiol. 2026 Sep 8;17:1893301. doi: 10.3389/fphys.2026.1893301 (PMC13593494; doi:10.3389/fphys.2026.1893301)
Supplement: Supplementary file 1 [file DataSheet1.docx]

Supplementary Table 1. Contents of key compounds at different time points in the air-breathing decompression group. Data are presented as median (interquartile range, IQR). The *P* values shown were obtained from the Friedman test.

| Compounds | Pre-exposure（ppb） | Mid-exposure（ppb） | Post-exposure（ppb） | *P*-value |
| --- | --- | --- | --- | --- |
| 1,2-Pentadiene | 471.564(0.159-658.623) | 444.85(0.389-817.702) | 871.915(0.369-1904.79) | 0.0232 |
| 1-Heptene | 2507.027(0.216-3747.274) | 3608.674(2.926-5318.397) | 2144.26(1.442-3176.596) | 0.0005 |
| 1-Pentene/(E/Z)-2-Pentene | 859.415(0.357-1092.238) | 873.109(0.772-1146.056) | 1686.347(0.966-2185.605) | 0.0085 |
| 2-Butanone | 1846.746(0.31-3398.12) | 1878.131(2.071-3178.729) | 741.523(0.882-1789.856) | 0.0071 |
| Carbon Disulfide | 106.42(0.062-144.603) | 170.538(0.085-278.089) | 114.526(0.073-149.766) | 0.0071 |
| Isoprene | 3138.38(0.916-3509.228) | 2402.902(1.734-2779.146) | 3866.369(3.648-4041.551) | 0.0001 |
| n-Heptane | 2285.772(0.491-2969.127) | 2982.036(2.741-4170.533) | 2192.6995(2.228-2442.469) | 0.0003 |
| Methylcyclohexane | 3927.258(0.6695- 5949.019) | 198.788(0.33- 568.019) | 106.874(0.255- 139.706) | 0.0140 |
| Acrolein | 254.042（0.132-318.473） | 211.795（0.237-279.033） | 298.877（0.325-575.359） | 0.0561 |
| Dimethyl Sulfide | 284.984（40.81025-705.63725） | 0.489（0.301-186.489） | 754.679（0.5945-1460.19475） | 0.0622 |

Supplementary Table 2. Contents of key compounds at different sampling time points in the oxygen-breathing decompression group. Data are presented as median (interquartile range, IQR). The *P* values shown were obtained from the Friedman test.

| Compounds | Pre-exposure（ppb） | Mid-exposure（ppb） | Post-exposure（ppb） | *P*-value |
| --- | --- | --- | --- | --- |
| 1-Pentene/(E/Z)-2-Pentene | 7622.479（5125.542-8799.576） | 1176.586（990.430-4898.326） | 8635.467（4895.814-9562.851） | 0.0015 |
| Acrolein | 418.526(339.488- 2446.298) | 311.132(266.20275- 365.988) | 1800.138(448.445- 2683.947) | 0.0103 |
| Acetone | 1526.698(1488.21375- 1604.60275) | 1225.9545(1050.23475- 1529.00475) | 1517.1465(1436.953- 1575.1575) | 0.0170 |
| Dimethyl Sulfide | 3329.313(3077.91875- 3498.6605) | 1553.296(257.35875- 2891.69275) | 3439.639(3375.877- 3669.537) | 0.00008 |
| Carbon Disulfide | 120.517(109.513- 139.402) | 87.136(66.9975- 109.9755) | 115.271 (100.287- 129.7895) | 0.0302 |
| (E/Z)-2-Butene | 2068.2585(1710.968- 2223.857) | 2193.893(724.893- 2882.895) | 2526.129(2260.252- 2880.552) | 0.0111 |
| Isoprene | 2634.1385(2431.132- 2750.864) | 1229.276(841.862- 2018.964) | 3237.589(2972.295- 3702.181) | 0.00007 |
| 2,2-Dimethylbutane | 3882.531（3825.413-3921.388） | 3480.995（2977.710-3937.085） | 3802.790(3719.583-3937.085） | 0.1092 |
| Methyl Acetate | 4210.010（3734.057-4581.904） | 3055.320（2322.407-3682.011） | 3055.320（2867.615-3645.464） | 0.0970 |

Supplementary Figure 1. Major VOCs of exhaled breath without significant change or trends in the content at different time points in diver experiencing decompression with air breathing. VOC concentrations were log₁₀-transformed for visualization, whereas statistical analyses were performed using the original concentration values.

**P* < 0.05 *vs* pre-exposure (Pre); ^#^ *P* < 0.05 *vs* mid-exposure (Mid); ^※^ *P* < 0.05 *vs* post-exposure (Post). Box plots depict medians (center lines), interquartile ranges (IQR, boxes), and 1.5 × IQR whiskers; outliers are shown as individual dots.

Supplementary Figure 2. Major VOCs of exhaled breath without significant change or trends in the content at different time points in divers experiencing decompression with oxygen-breathing. VOC concentrations were log₁₀-transformed for visualization, whereas statistical analyses were performed using the original concentration values.

**P* < 0.05 *vs* before exposure (Pre); ^#^*P* < 0.05 *vs* immediately before decompression (Mid); ^※^*P* < 0.05 *vs* after exposure (Post). Box plots depict medians (center lines), interquartile ranges (IQR, boxes), and 1.5 × IQR whiskers; outliers are shown as individual dots.
